# Comparative Analysis of CT Fluoroscopy Modes and Gastropexy Techniques in CT-Guided Percutaneous Radiologic Gastrostomy

**DOI:** 10.3390/tomography10110129

**Published:** 2024-11-06

**Authors:** Michael P. Brönnimann, Mauro Tarca, Laura Segger, Jagoda Kulagowska, Florian N. Fleckenstein, Bernhard Gebauer, Uli Fehrenbach, Federico Collettini, Johannes T. Heverhagen, Timo A. Auer

**Affiliations:** 1 Department of Diagnostic, Interventional and Pediatric Radiology, Inselspital, Bern University Hospital, University of Bern, Freiburgstrasse 10, 3010 Bern, Switzerland; jagoda.kulagowska@insel.ch (J.K.); johannes.heverhagen@insel.ch (J.T.H.); 2 Department of Radiology, Charité—Universitätsmedizin, Berlin, Augustenburger Platz 1, 13353 Berlin, Germany; laura.segger@charite.de (L.S.); florian.fleckenstein@charite.de (F.N.F.); bernhard.gebauer@charite.de (B.G.); uli.fehrenbach@charite.de (U.F.); federico.collettini@charite.de (F.C.); timo-alexander.auer@charite.de (T.A.A.); 3 Faculty of Medicine, University of Bern, Murten Street, 3008 Bern, Switzerland; mauro.tarca@students.unibe.ch; 4 Clinician Scientist Program, Berlin Institute of Health at Charité—Universitätsmedizin Berlin, 10178 Berlin, Germany

**Keywords:** gastrostomy, radiation dosage, radiography, interventional, tomography, operative time, treatment outcome, head and neck neoplasms

## Abstract

Background/Objectives: This study was conducted to compare two modes of computed tomography fluoroscopy (CTF) and two gastropexy techniques used in CT-guided percutaneous radiologic gastrostomy (CT-PRG) aiming to identify the optimal techniques for image guidance and gastropexy and, thus, to overcome the current lack of consensus on the preferred modalities. Methods: We retrospectively identified 186 successful CT-PRG procedures conducted evenly across two university hospitals from January 2019 to December 2023. Patients were divided into two groups (intermittent multislice CT biopsy mode-guided technique (MS-CT BM) and retention anchor suture (T-fastener) versus real-time (RT-)CTF and gastropexy device) for descriptive analysis of demographics, indication for PRG, radiation exposure (DLP), procedural time, number of CT scans, gastropexy time, and complications. Differences were assessed for statistical significance using Fisher’s exact test and the Mann–Whitney U-test. Results: Our final study population comprised 100 patients (50 from each center; 62.52 ± 12.36 years, 73 men). There was a significant difference in radiation exposure between MS-CT BM (group 1) and RT-CTF (group 2), with an average dose-length product (DLP) of 56.28 mGycm×m ± 67.89 and 30.91 ± 27.53 mGycm×cm, respectively (*p* < 0.001). PRG with RT-CTF guidance was significantly faster than PRG with MS-CT BM, with an average difference of 10.28 min (*p* < 0.001). No significant difference in duration was found between the two gastropexy methods compared (retention anchor suture, 11.50 ± 5.239 s vs. gastropexy device, 11.17 ± 6.015 s; *p* = 0.463). Complication rates did not differ significantly either (*p* = 0.458). Conclusions: Our findings indicate comparable efficacy and safety of the two gastropexy methods and underscore that the choice of CTF mode for image guidance has only a small role in reducing radiation exposure in patients undergoing CT-PRG. Instead, it is essential to avoid control scans.

## 1. Introduction

Patients with tumor-induced or neurogenic swallowing impairment often require enteral tube feeding, which offers both physiologic and economic advantages over parenteral nutrition in patients requiring extended care [1]. Tubes can be inserted using percutaneous radiologic gastrostomy (PRG) with image guidance by fluoroscopy (F-PRG), the traditional method, or computer tomography (CT), which is increasingly being used and has significantly lower rates of minor (F-PRG, 7.8–23% vs. CT-PRG, 7.7%) and major complications (5.9–10% vs. 4%) [1,2,3,4,5,6,7,8,9] at the cost of a longer procedure time (F-PRG, 25.57 ± 5.99 min vs. CT-PRG 45.47 ± 8.98 min) and significantly higher radiation dose [10].

CT fluoroscopy (CTF) for image guidance is performed in one of two modes: continuous (real-time) or intermittent (quick check) [11]. Continuous CT fluoroscopy (RT-CTF) enables real-time imaging and frequently exposes the operator to a higher occupational radiation dose, because parts of his body, such as his arm, remain directly in the beam path during image acquisition. Conversely, the multislice CT biopsy mode-guided technique (MS-CT BM) involves intermittent axial acquisition for sequential guidance of the advancement of the access device into the target region. The operator adjusts the access device based on the acquired images until the target position has been reached [11,12,13]. To date, only Prosch et al. [13] have demonstrated through direct comparison during lung biopsies that MS-CT BM results in a significant 10-fold reduction in radiation dose compared to CTF (*p* < 0.001) but significantly longer procedure time (*p* = 0.04). The transferability of these results to another anatomical region remains unclear.

Gastropexy is another tool that can prevent immediate major complications by promoting adhesion formation, reducing the risk of initial intraperitoneal tube placement, and preventing subsequent tube migration [14,15,16]. It uses a retention anchor suture (T-fastener) or a gastropexy device. To the best of our knowledge, only one study has directly compared these two fixation methods for percutaneous endoscopic gastrostomy (PEG), demonstrating a higher rate of minor complications with the T-fastener anchors (10/26, 38% vs. gastropexy device, 6/26, 23%) [17]. In general, published data suggest that the incidence of minor (9.5–11.1%) and major (0.5–1.9%) complications associated with the T-fastener anchor method, when two or more anchors are placed, is notably lower [14]. However, data are sparse, and results may vary with the inclusion criteria used. Nothing is known about the impact on procedural time.

Furthermore, there is currently no consensus regarding the best methods for CTF image guidance and gastropexy in patients undergoing radiologic gastrostomy. This retrospective study seeks to address these gaps by directly comparing the efficacy, safety, and radiation exposure of two CTF modes and gastropexy techniques, aiming to refine current practices and improve clinical outcomes for both the patients and the medical personnel involved in gastrostomy procedures. In addition, our primary goal is to develop strategies that reduce both patient radiation exposure and procedure time during CT-guided PRG. With the anticipated increase in the use of this procedure due to its superior safety profile and lower complication rates compared to alternative techniques, these improvements are crucial for optimizing patient care.

## 2. Materials and Methods

**Figure 1:**
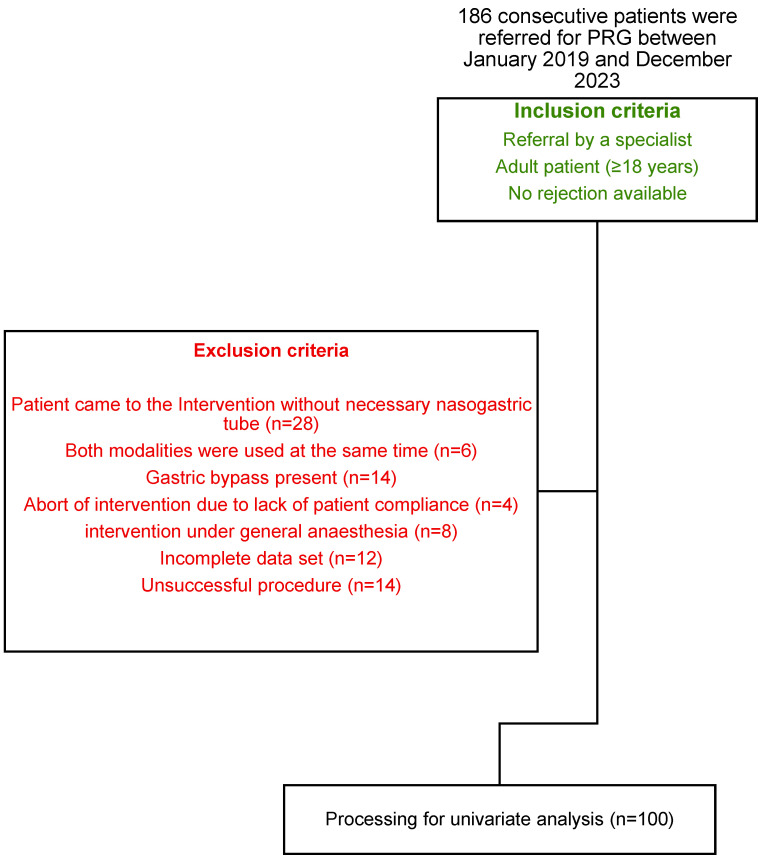
Flowchart showing the study population.

For our approach, we initially identified a total of 186 PRGs performed in equal parts at two university hospitals from January 2019 to December 2023: Inselspital, Bern University Hospital (group 1) and Charité—Universitätsmedizin Berlin, Campus Virchow Klinikum (group 2). Our retrospective study was approved by the ethics committees of the Canton of Bern (registration number: BASEC ID 2024-00772) and the State of Berlin (EA4/087/21). It was conducted in accordance with the principles of the Declaration of Helsinki. Written informed consent was obtained from all patients for the procedure and the use of their data for scientific purposes. Factors that significantly influenced procedure time were ruled out in order to achieve as accurate a comparison as possible between the two groups (Figure 1). Procedure time was defined as the time difference between the first and last images.

### 2.1. Baseline Assessment and Preparatory Measures

Patients were ideally informed 24 h prior to the intervention, and their medical histories and relevant blood parameters were reviewed. All patients were required to fast for 6 h, and a nasogastric tube was inserted by the ward doctor before the procedure. Board-certified independent interventional radiologists conducted the interventions, with a radiographer and an interventional radiology nurse present throughout the procedure. Patients were positioned supine on the table, and room air was administered through the nasogastric tube using a luer lock syringe and a three-way stopcock. Preprocedural sterilization of the skin was performed using 10% povidone-iodine, and local anesthesia with 1% lidocaine was administered subcutaneously and deeper into the stomach wall before each necessary step.

### 2.2. CT-Guided PRG Techniques at the Two Study Sites

#### 2.2.1. Group 1 (MS-CT BM and Retention Anchor Suture)

**Figure 2:**
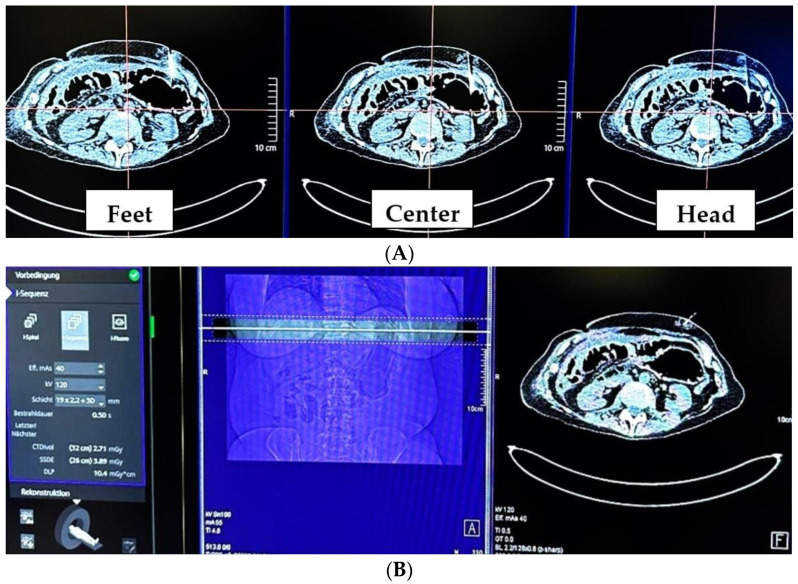
MS-CT biopsy mode (**A**) This screenshot was captured during an MS-CT biopsy mode-guided procedure, showing the image output used by the interventionalist to plan the next steps. When activated by the radiographer using the foot pedal, the CT scanner acquires and displays images of three adjacent sections. The upper image corresponds to the level of the gantry laser beam visible on the patient’s surface. The anchor needle runs from bottom to top. (**B**) Screenshot of the planning console. Patient positioning and scan area are visible. In addition, the acquired images can also be viewed one by one.

Procedures were performed on a Somatom X.cite CT scanner (Siemens Healthineers, Erlangen, Germany). The responsible interventionalist ensured adequate gastric filling based on the CT scout view, which was followed by the acquisition of an unenhanced upper abdominal CT scan covering the target region and reconstructed at 1 mm increments. The standardized intervention included the multislice CT biopsy mode-guided technique (MS-CT BM) i-Sequence with the following parameters: 120 kV, 25 mA s, a rotation time of 0.5 s, and a range of 12 mm with three slices of 4 mm. Upon activation by the interventionalist standing next to the gantry, three contiguous slices were immediately captured, facilitating needle position verification and allowing adjustments if necessary (Figure 2).

**Figure 3:**
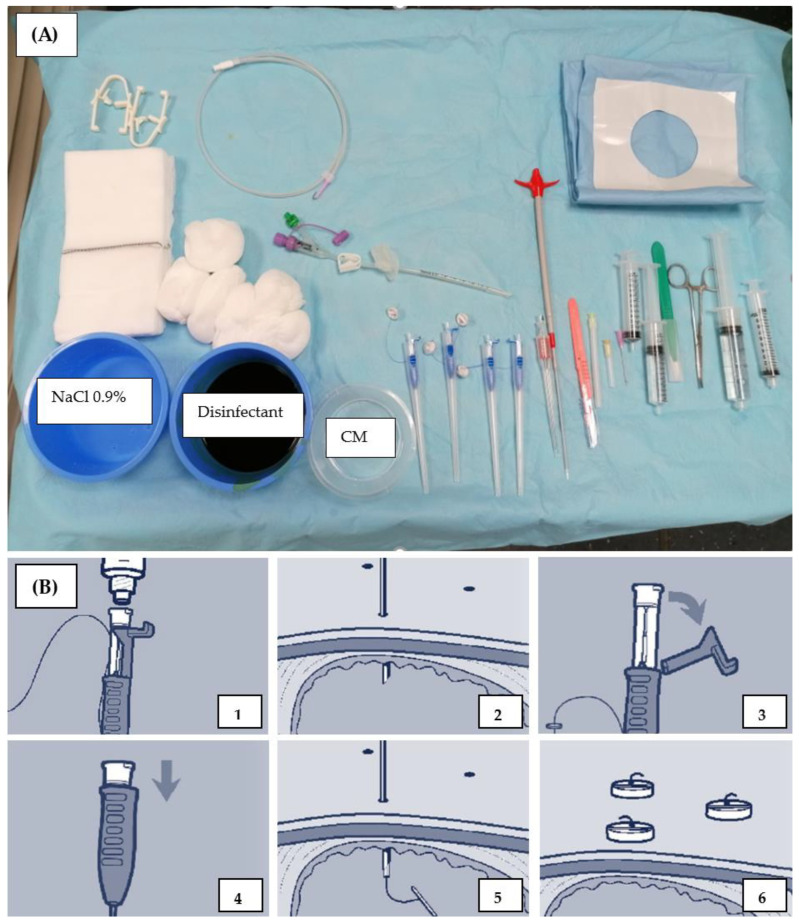
Intervention material used at Inselspital University Hospital Bern, Switzerland. (**A**) Avanos Introducer Kit for Gastrostomy Feeding Tube with four SAF-T-PEXY T-Fasteners next to CM (contrast media) and 10 mL Iopamiro 300 diluted with sodium chloride with a ratio 1:2 (Bracoo Suisse SA, Cadempino, Switzerland). Flocare^®^ Gastro Tube CH 14 in the middle. The 0.035” J-tip guidewire is above the gastric tube. The Avanos Enteral Access Dilation System is left to the pink scalpel, and Lidocain 1% is in the bigger syringe. In the other syringe, there is 10 mL sterile water, Aqua ad iniectabilia Bichsel (Large pharmacy Dr. G. Bichsel AG, Interlaken, Switzerland). NaCl = sodium chloride. (**B**) Schematic illustration of the use of SAF-T-PEXY T-Fastener. (1) Attach a luer slip syringe containing 1–2 mL of sterile water or saline to the needle hub. (2) Insert the preloaded SAF-T-PEXY slotted needle with a single sharp thrust through one of the marked corners of the triangle until it reaches the gastric lumen. The simultaneous return of air into the syringe and visualization confirms the correct placement. (3) Release the suture strand and bend the locking tab on the needle hub. (4) Firmly push the inner hub into the outer hub until the locking mechanism clicks into place. (5) This will release the T-bar from the end of the needle and lock the inner stylet into position. (6) Withdraw the needle while gently pulling the T-bar until it lies flush against the gastric mucosa. Avoid applying excessive tension on the T-bar against the gastric mucosa. Discard the needle following facility protocol [18].

Adaptive iterative dose reduction using three-dimensional processing (AIDR3D STD) was employed to minimize radiation exposure. No breathing commands were given to the patient based on our experience that patients tended to overcompensate afterward, resulting in longer procedures because subsequent CTF scans failed to adequately cover the target range. Each needle and guide wire position was verified by MS-CT BM, with the patient repositioned out of the gantry for each action. In case of uncertainty or if the needle was not visible due to strong patient respiratory movement or decreasing filling level at any point, a control CT was conducted. The Avanos introducer kit was used for PRG insertion (Figure 3).

**Figure 4:**
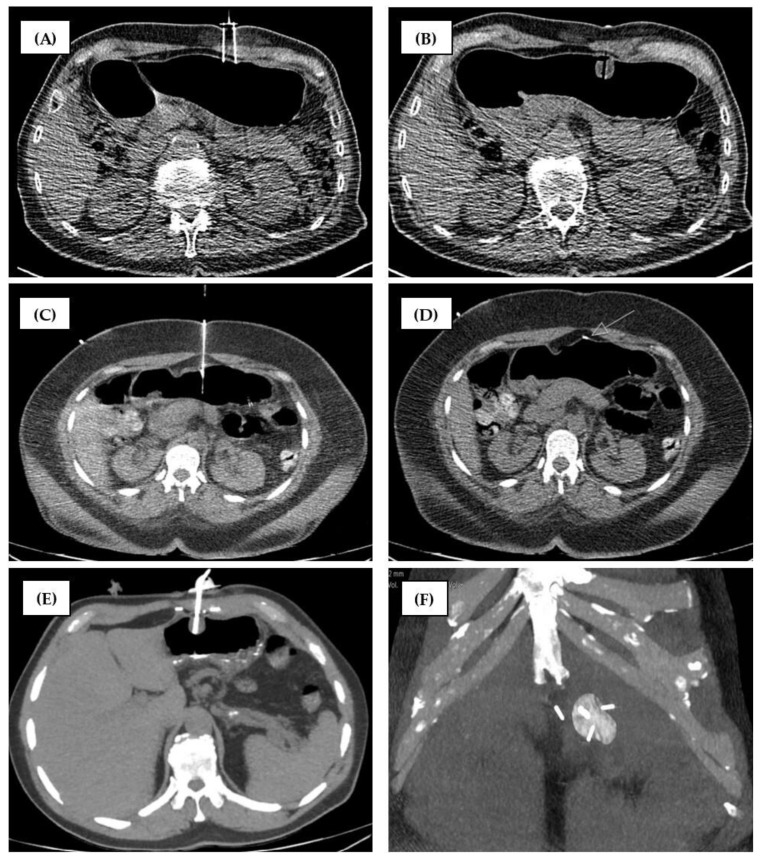
Comparison of fixation techniques. (**A,B**) technique of center 2. (**C–F**) technique of center 1. (**A**) Gastropexy Device II (Fresenius Kabi, Bad Homburg vor der Höhe, Germany) with two parallel hollow needles and a thread inside. (**B**) After gastropexy, the suture is not visible on CT. (**C**) A first SAF-T-PEXY T-Fastener anchor (Avanos Medical, GA, USA) was placed in the body of the stomach. (**D**) The anchor is visible in the CT. The arrow shows the correct end position on the inner stomach wall. (**E**) Gastric tube placed between the anchors. (**F**) The distances between the anchors are different. As usual, the tube was placed in the center of the triangle.

A maximum of three gastropexies (2 cm apart, retention anchor suture, ideally in triangle configuration) were performed before wire insertion through an 18G needle, successive dilation, and tube insertion using the over-the-wire push-type method (Figure 4C–F). Air was administered (6 × 50 mL) just before gastric tube insertion to generate sufficient counterpressure.

Subsequently, a final upper abdominal CT scan was performed after balloon blocking with 5 mL sterile water and luminal contrast agent administration (10 mL Iopamiro 300 mg/mL; lopamidolum; Bracco Suisse SA, Cadempino, Switzerland, diluted with sodium chloride at a 1:2 ratio). Additionally, the wound was covered with a metalline^®^ aluminum vaporized coating compress.

#### 2.2.2. Group 2 (RT-CTF and Gastropexy Device)

**Figure 5:**
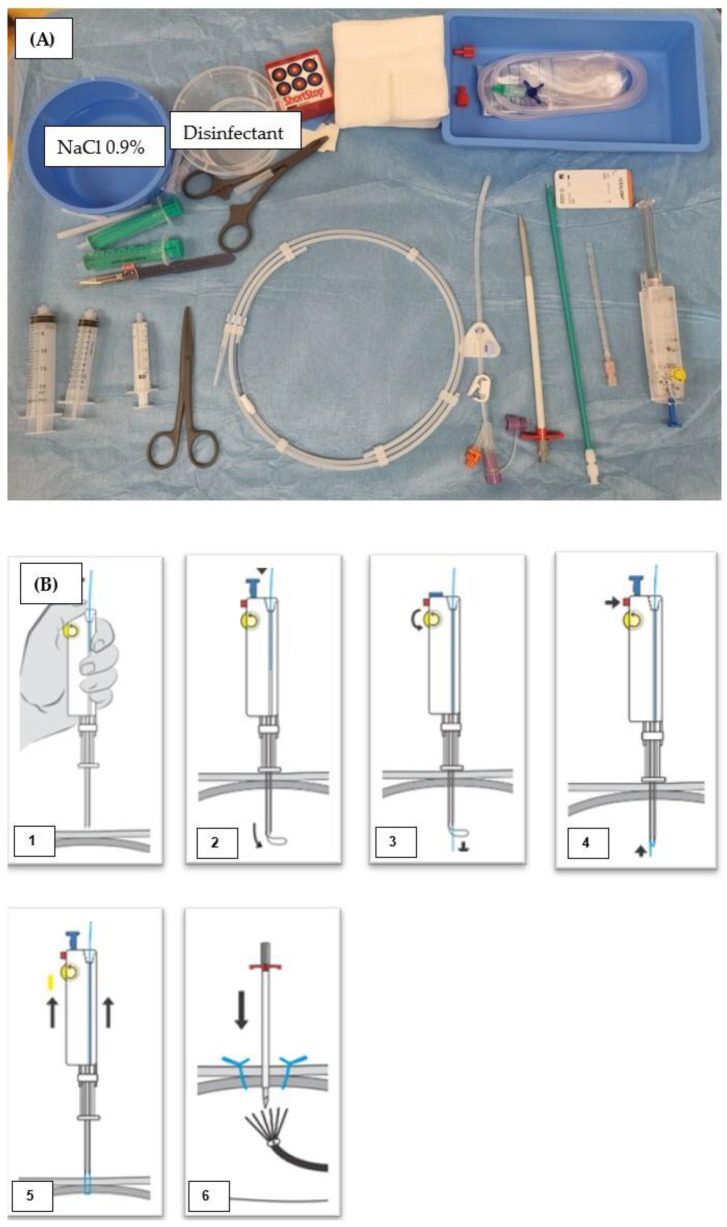
Intervention material used at Charité, University Hospital Berlin, Germany. (**A**) In the bottom row, from right to left: the Freka Pexact-Set CH 15 II (Fresenius Kabi, Bad Homburg vor der Höhe, Germany) with Gastropexy Device II, the puncture needle 18 gauge, the dilator in green 12 French, the 16 French peel-away sheath with dilator inside, the Freka^®^ GastroTube CH 15, the 0.035″ 75 cm Amplatz guidewire (Merit Medical GmbH; Limburg an der Lahn, Germany), Lidocain 1% in the bigger syringe, and 10 mL sterile water in the other syringe (Aqua ad iniectabilia Bichsel (Large pharmacy Dr. G. Bichsel AG, Interlaken, Switzerland)). NaCl (sodium chloride). (**B**) Schematic illustration of the use of Gastropexy Device II. (1) Insert the thread, followed by the gastropexy device. (2) Push the insertion rod to open the loop wire. (3) Pass the thread through the loop wire. (4) Press the release button to close the loop wire and secure the thread. (5) Remove the gastropexy device and tie a knot. (6) Insert the trocar [19].

Procedures were performed on a Somatom Definition AS CT scanner (Siemens Healthineers, Erlangen, Germany). The standardized intervention involved the use of real-time computed tomography fluoroscopy (RT-CTF) with the following parameters: 100 kV, 40 mA s, a rotation time of 0.36 s, and a 1 × scanning length of 5 mm. The interventionalist stayed in the room throughout the procedure, and each intervention step started by triggering the CT scanner using the foot pedal. The single images were displayed in real time on the monitor. The Freka Pexact-Set CH 15 II was used for PRG insertion (Figure 5).

Two gastropexies 2–4 cm apart were performed with sutures. The anterior gastric wall was percutaneously punctured with a gastropexy device, which consists of two parallel needles and a loop (Loop Fixature, Gastropexy Device II, Fresenius Kabi, Bad Homburg vor der Höhe, Germany) (Figure 4A,B). A manual knot at skin level fixed the suture-based gastropexy. The patient was only moved out of the gantry if the instruments could not otherwise be handled in the confined space. The gastric tube was inserted using the Seldinger technique after prior successive dilatation with several dilators (12–16 F, Cook Germany GmbH, Mönchengladbach, Germany) and via the 16 F peel-away sheath. A final upper abdominal CT scan was performed after balloon blocking with sterile water (5–7.5 mL). A slit compress was positioned under the gastric tube to cover the wound and was tightened at the end of the procedure.

### 2.3. Follow-Up

Patients’ vital signs and overall condition were continuously monitored in the ward for 6 h after the interventions. No peri- or post-procedural antibiotics were administered. The patients fasted for 6 h before nutrition via the tube was initiated and then slowly increased. By the next day, the nasogastric tube was removed. Medical and surgical teams continued to provide follow-up care for three months, including assessments for complications, adequacy of feeding support, and tube outcomes. Any issues or malfunctions identified during follow-up were referred to the interventional radiology department. Patients experiencing tube-related problems were advised to contact the radiology department directly via a 24 h telephone number provided in the nursing care booklet handed to each patient after the procedure. The stitches of the suture (anchors) were trimmed to skin level by the family doctor after 5–7 days, and routine tube replacement was recommended after 3 months. A detailed overview of the different settings at the two study centers is given in Table 1.

### 2.4. Data Evaluation

Two board-certified independent interventional radiologists with eight years of experience who did not conduct any of the interventions reviewed all procedures. In cases where they disagreed, a consensus was reached through discussion. If consensus could not be achieved, a third experienced radiologist was consulted to make the final decision. Patients were categorized into two groups. We collected data on patient demographics, indication for PRG insertion, radiation exposure parameters (dose-length product (DLP)), procedural time (in minutes), number of acquired CT scans, gastropexy time, and complications. Procedural time was defined as the duration from the acquisition of the first to the last images stored in the picture archiving and communication system (PACS). Gastropexy time (GT) was defined as the interval between the first and the last image in which the devices were visible. Information pertinent to the intervention, including indications and complications, was retrieved from the electronic patient files covering the period from one day before insertion to three months after insertion (during regular tube replacement). Complications were categorized and defined as minor (e.g., superficial wound infection, minor leakage, dislodgment, or abdominal wall hematoma) and major (e.g., hemorrhage, peritonitis, profound wound infection, perforation/tear, aspiration, tube displacement, or sepsis) using the criteria outlined by Kandarpa et al. [20]

### 2.5. Statistical Analysis

Patient characteristics and interventional parameters were compared using the Mann–Whitney U-test for continuous variables and Fisher’s exact test for categorical variables. The Kolmogorov–Smirnov test was applied to assess for normal distribution. Statistical analysis was performed with commercially available software (IBM SPSS Statistics for Windows, version 28; IBM, Armonk, NY, USA). All tests were performed in a two-sided fashion with a level of significance of *p* < 0.05.

## 3. Results

### 3.1. Study Population

Our final study population included 100 patients enrolled after the application of our inclusion/exclusion criteria (Figure 1). The two groups did not differ significantly in terms of age and sex distribution. All variables were not normally distributed in both groups according to the Kolmogorov–Smirnov test (*p* < 0.001). Almost three-quarters of the patients were men. The mean age was just over 60, with a range of 26 to 84 years (Table 2). Head and neck cancer accounted for the majority of indications for PRG in our study population (70% in group 1 and 90% in group 2). There was a significant difference in this category due to the higher proportion of patients with neurologic disease in group 1 (12/50, 24%) compared with group 2 (2/50, 4%; *p* = 0.011).

### 3.2. Interventional Parameters

Despite an approximately three times longer exposure time (5.88 s compared to 16.69 s, *p* < 0.001), the resulting CT tube radiation exposure was almost half as large (average DLP of group 1 56.28 mGycm×cm ± 67.89 vs. group 2 30.91 ± 27.53 mGycm×cm, *p* < 0.001). The significant difference in the number of CT scans (mean of 3.42 vs. 1.2, *p* < 0.001) with a proportional share to total DLP of 0.12 and 0.14, respectively, (*p* = 0.011) led to a significant difference in total DLP of the two image guidance modes investigated (681 mGy×cm vs. 244, *p* < 0.001). No significant difference in duration was found between the two gastropexy techniques (*p* = 0.463), while total procedure time was, on average, significantly shorter for CT-PRG with RT-CTF (group 2) than for PRG with MS-CT BM. The difference was 10.28 min on average (*p* < 0.001). An average of 2.77 anchors (n = 1, 3, 6%; n = 2, 15, 30%; n = 3, 22, 44%; n = 4, 10, 20%) were placed in group 1.

### 3.3. Outcome

In terms of major complications, there was one case of peritonitis in group 1 (in a patient with placement of one anchor) and a relevant hemorrhage in group 2 (each 2%). The most common minor complication in both groups was earlier tube dislodgment before the first regular change (3/7, 6% and 6/12, 12%). There were two cases of superficial wound infection in group 2 (2/12, 4%) and none in group 1 (0/7, 0%). Gastrostomy leakage occurred once in group 2 (1/12, 8.3%) but never in group 1 (0/7, 0%), and no clinically relevant pneumoperitoneum was reported in either group. The difference in overall incidence of complications was not statistically significant between the two groups (*p* = 0.458) (Table 2).

## 4. Discussion

This two-center study presented here aimed at investigating and comparing the effectiveness, safety, and radiation exposure associated with intermittent versus real-time CTF and two gastropexy methods in CT-guided PRG. The methods were retrospectively compared in a total of 100 patients. Gastropexy time (*p* = 0.463) and complications (*p* = 0.458) did not differ significantly between the two groups. Head and neck cancer was by far the most common indication for PRG in both groups, although there was a higher proportion of patients with neurologic diseases in group 1 (*p* = 0.011). The total and CTF-specific radiation exposure was notably higher when the MS-CT BM mode was used for image guidance during CT-PRG procedures (56.28 ± 67.897 vs. 30.91 ± 27.539 mGycm×cm, *p* < 0.001), and the interventions took longer (41.08 ± 16.97 vs. 30.40 ± 23.83 min, *p* < 0.001). Regardless of the mode, CTF accounted for only 14–18% of the total DLP. These findings suggest that the image guidance mode alone does not lead to a substantial radiation exposure reduction in CT-PRG. Moreover, the two gastropexy methods we investigated and compared are comparable in terms of efficacy and safety in our study population.

Although mean exposure time was significantly shorter, by about one-third (5.88 ± 5.618 vs. 16.69 s ± 15.183, *p* < 0.001), average radiation exposure in MS-CT BM was 82% higher (56.28 ± 67.897 vs. 30.91 ± 27.539 mGycm×cm, *p* < 0.001). This is due to the larger scan area of 3 × 4 mm vs. 5 mm and the higher tube voltage (120 kV vs. 100 kV). The radiation dose varies with the square of the tube voltage [21]. Given the notably higher number of patients with neurologic diseases in group 1 (12/14, 24% vs. 2/13, 4%, *p* = 0.011) and the exclusive use of local anesthesia, there may have been a selection bias contributing to the larger number of control CT scans. Additionally, intermittent CTF is controversial in this anatomic region due to motion artifacts. According to Carlson et al., the use of the intermittent mode can be a major problem during interventions in the lungs or the upper abdomen [11]. Nevertheless, CTF was found to account for only 12–14% of the total radiation exposure during CT-PRG. This aligns with the conclusions of our previous study [22] and the findings reported by Nattenmüller et al. [23], who found that the additional exposure to ionizing radiation from CT-guided interventions was, on average, 2.6 mSv (effective dose). They concluded that postinterventional control CT scans should only be recommended for retroperitoneal interventions. Consequently, regardless of the selected CTF mode, CT-PRG should be attempted without helical CT scans. The gastric filling status and initial positioning ought to be ascertained through the CT scout view. This could be an interesting approach that deserves further investigation in future studies. In addition, the radiation exposure of the staff performing CT-PRG procedures with RT-CTF should not be forgotten in this context [24].

Average procedure times were 41.08 ± 16.97 min for CT-PRG with intermittent CTF and 30.40 ± 23.83 min for procedures with RT-CTF, corresponding to a significant difference of 10.28 min (*p* < 0.001). These findings are consistent with published data. Hu et al. [8] found a mean procedure time of 45.47 ± 8.89 min for CT-PRG. Tamura et al. [4] reported a mean procedure time of 25.3 min for CTF-PRG with a gastropexy device. Furthermore, our findings corroborate the results of Prosch et al. [24], who found procedures with RT-CTF to be faster than procedures with MS-CT BM. The use of different gastropexy techniques alone cannot explain the significant time difference (*p* = 0.463). One factor that likely influenced this outcome is the unique anatomic conditions previously discussed in relation to intermittent CTF.

The common minor complication we encountered, tube dislodgment, occurred in 18% of all cases (6% in group 1 and 12% in group 2), consistent with the findings of de Baere et al. (10.2%) [16], Yang et al. (22.4%) [6], and Lorentzen (14%) [3]. Our experience furthermore aligns with de Baere’s observation of additional challenges in maintaining the tube and the risk of balloon perforation when expandable balloon gastrostomy tubes are used. To mitigate this problem, we labeled the balloon catheter of the Flocare^®^ Gastro Tube CH 14 with a red sticker reading “Do not use—balloon access warning!”. This measure significantly reduced this complication. The risk of tube dislodgment is minimized with the Freka^®^ GastroTube CH 15 due to its incompatible balloon inflation port with a luer-lock syringe. However, the recommended sterile water volume for balloon inflation may be insufficient for longer dwell times, especially for larger probes like CH 18 [25]. Further investigation of the relationship between balloon filling volume and dislodgment rate is warranted, potentially in collaboration with manufacturers. The incidences of gastropexy-related minor complications like stomal leakage, peristomal superficial infection, and pneumoperitoneum were also in line with previously published data. A total of 2% superficial wound infections and 1% leakages occurred (compared with 1% and 0.2% in [16]). Perhaps the use of metalline^®^ aluminum vaporized coating compresses can explain the difference between the two groups in our study. With a 3% of major complications, our results are in line with the sparse data reported in the literature (e.g., 4% in the study of Tamura et al. [4]). The occurrence of peritonitis as a major complication with the use of only one anchor in a patient of group 1 is likely not coincidental, considering the existing literature [14]. The lower incidence of major complications associated with the use of the gastropexy device in group 2 compared to the results reported by Gang et al. [17] (2% vs. 23%) may be explained by the fact that they did not use the metallic trocar for insertion.

Our study has several limitations. First, it was conducted as a retrospective analysis of a relatively small number of patients. Second, in order not to compromise the primary endpoint, only successful CT-PRGs were included. Third, an important consideration for this interventional procedure is that the effective radiation dose to the interventionalist was not measured. Future prospective studies should address this.

## 5. Conclusions

Our findings indicate comparable efficacy and safety of the two gastropexy methods investigated and underscore that the choice of intermittent versus real-time CTF does not make a relevant contribution to radiation exposure reduction in CT-PRG. Continued endeavors are essential to further minimize both radiation exposure and procedure duration in CT-PRG. Furthermore, we conclude that by eliminating control scans (relying solely on the CT scout view for insertion site planning and RT-CTF for guidance) and optimizing patient preparation (e.g., pre-placement of the nasogastric tube in the ward) and team coordination (e.g., clear task delegation such as air administration and assistance), CT-guided PRG insertions are likely to become the preferred method in the future. This is due to their significantly lower risk of complications compared to other modalities, making them a superior option for patient safety. The choice of fixation technique is of secondary importance in this context.

## Figures and Tables

**Table 1:** Comparison of settings. CTF = computed tomography fluoroscopy; INR = international normalized ratio; Hb = hemoglobin; MS-CT BM = multi-slice computed tomography biopsy mode; RT = real-time; F = French; h = hours; aPTT = activated partial thromboplastin time.

	Center 1	Center 2
**Staff present**	1 × interventionalist, radiographer, interventional radiology nurse	
**Preparation**		
Required lab values	INR ≤ 1.5, Quick ≥ 60%, Hb ≥ 80g/L, platelate value ≥ 50 × 10^9^/L	INR ≤ 1.5, Quick ≥ 50%, aPTT < 50 s, platelate value ≥ 50 × 10^9^/L
Drugs during intervention	Lidocaine 1% (max. 20 mL), 20 mg Buscopan	Lidocaine 1%, 5 mg Midazolam, 8 mg Ondansetron and 15 mg Piritramid, if necessary (Buscopan 20 mg)
Stomach insufflated volume	600 mL (room air)	800 mL (room air)
**Scanner**		
Type	Toshiba Asteion 4SL/Somatom X.cite	Somatom Definition AS
Mode	MS-CT BM	RT-CTF
Name	i-Sequence	i-Fluoro
CTF	intermittent	continuous
Acquired slices	3 × 4 mm	1 × 5 mm
Tube settings	120 kV, 25 mAs, rotation time 0.5 s	100 kV, 40 mAs, rotation time 0.36 s
**Operator experience**	board-certified radiologist and at least one year of experience in this procedure	
**Material**		
Gastric tube	Flocare^®^ Gastro Tube CH 14	Freka^®^ Pexact CH/FR 15
Fixation material	4 × single anchors	gastropexy device
Fixation technique	Free choice of anchor number, min 1.	4 point with 2 sutures, distance to each of 2 cm
Dilatation	Enteral Access Dilation System 12–16 F	several, single dilatators 10, 12 and 16 F
Additional material	metalline^®^ aluminum vaporized coating compress	Slit compress
**Start of nutrition**	early feeding 6 h after intervention	

**Table 2:** Univariate analysis of CT-guided PRG grouped by modality with patient demographics, interventional parameters, and complications. Unless stated otherwise, data are the number of biopsies ± standard deviations. ×2 (R × 2), Fisher’s exact test, and Mann–Whitney-U-Test were used to calculate the statistical difference between groups of categorical, dichotomous, and continuous variables, respectively. Data are mean ± standard deviation. * statistically significant (defined by *p* < 0.05); y = year; mGy = milliGray; s = seconds; No = number; GT = gastropexy time; DLP = dose length product; C = center; MS-CT BM = multislice computed tomography biopsy mode; RT-CTF = real-time computed tomography fluoroscopy.

Survey of PRG							
Parameter	All (*n* = 100)		C1, MS-CT BM with Single Anchor (*n* = 50)		C2, RT-CTF with Gastropexy Device (*n* = 50)		*p* Value
**Female**	27	27%	14	28%	13	26%	1.000
**Age (y)**	62.52	±12.36	62.52	±12.36	64.18	±12.44	0.288
**Indication**							0.011 *
HN-cancer	80	80%	35	70%	45	90%	
Neurological disorders	14	14%	12	24%	2	4%	
Other indications	6	6%	3	6%	3	6%	
**DLP of CTF (mGy×cm)**	43.59	±53.10	56.28	±67.897	30.91	±27.539	<0.001 *
**Exposure Time (s)**	11.63	±12.86	5.88	±5.618	16.69	±15.183	<0.001 *
**Proportion of CTF DLP to total DLP**	0.13	±0.12	0.12	±0.134	0.14	±0.111	0.011 *
**No. of CT**	2.31	±1.71	3.42	±1.797	1.20	±0.404	<0.001 *
**DLP of CTs (mGy×cm)**	390.43	±545.82	582.37	±714.3	198.50	128.54	<0.001 *
**Total DLP (mGy** **×** **cm)**	462.90	±582.92	681.00	±751.49	244.62	±156.74	<0.001 *
**GT (min)**	11.14	±5.725	11.50	±5.239	11.17	±6.015	0.463
**Procedure time (min)**	35.74	±21.27	41.08	±16.97	30.40	±23.83	<0.001 *
**Complications**							0.458
Minor	19	19%	7	14%	12	24%	
Major	2	2%	1	2%	1	2%	

## Data Availability

Dataset available on request from the authors.
